# Impact of Adrenomedullin on Mitochondrial Respiratory Capacity in Human Adipocyte

**DOI:** 10.21203/rs.3.rs-2600140/v1

**Published:** 2023-03-06

**Authors:** Yuanlin Dong, Vidyadharan Alukkal Vipin, Chellakkan Selvanesan Blesson, Chandrasekhar Yallampalli

**Affiliations:** Baylor College of Medicine; Baylor College of Medicine; Baylor College of Medicine; Baylor College of Medicine

## Abstract

For metabolic homeostasis adequate mitochondrial function in adipocytes is essential. Our previous observation showed that circulating levels of adrenomedullin (ADM) and mRNA and protein for ADM in omental adipose tissue were higher in patients with gestational diabetes mellitus (GDM) compared with normal pregnancy, and these alterations are accompanied by glucose and lipid metabolic dysregulation, but the impact of ADM on mitochondrial biogenesis and respiration in human adipocyte remain elusive. In this study we demonstrated that: (1) Increasing doses of glucose and ADM inhibit human adipocyte mRNA expressions of mitochondrial DNA (mtDNA)-encoded subunits of electron transport chain (ETC), including nicotinamide adenine dinucleotide dehydrogenase (ND) 1 and 2, cytochrome (CYT) b, as well as ATPase 6; (2) ADM significantly increases human adipocyte mitochondrial reactive oxygen species (ROS) generation and this increase is reversed by ADM antagonist, ADM22–52, but does not significantly affect adipocyte mitochondrial contents; (3) Adipocyte basal and maximal oxygen consumption rate (OCR) are dose-dependently suppressed by ADM, and results in impaired mitochondrial respiratory capacity. We conclude that elevatedADM observed in diabetic pregnancy may be involved in glucose and lipid dysregulation through compromising adipocyte mitochondrial function, and blockade of ADM actions in adipocytes may improve GDM-related metabolic complications.

## Introduction

Adipose tissue is not only an energy reservoir for lipid droplets, but also an important endocrine organ, secreting pro-inflammatory cytokines, reactive oxygen species, and adipokines, including adrenomedullin (ADM)^1,2^. Adipose tissue dysregulation contributes to the pathophysiology of a variety of metabolic disorders, including cardiovascular diseases, obesity, polycystic ovary syndrome (PCOS), and diabetes mellitus^3,4^ Recent studies in the rat model show that ADM and its receptors are expressed in adipose tissue^5^, administration of ADM induces hyperglycemia, which can be reversed by an ADM neutralizing antibody^6^. In humans, plasma ADM concentrations are elevated in obese individuals^7^ and patients with T2DM^8^. Our previous studies have shown that both circulating ADM and mRNA and protein of ADM in omental adipose tissue were increased in GDM patients^9,10^, indicating the involvement of ADM in the impaired metabolic homeostasis. However, the underlying mechanisms of ADM contributing to GDM-related metabolic dysregulation remain unclear.

Emerging evidence indicated that mitochondria play central roles in energy homeostasis, metabolism, pathway signaling, and cellular apoptosis^11^, and mitochondrial dysfunction in adipocytes is tightly related with insulin resistance in obese and diabetic individuals^12,13^. Patients with type 2 diabetes show that mitochondrial functions are declined, which are associated with a reduction of both mitochondrial DNA (mtDNA) copy numbers and key factors regulating mitochondrial biogenesis^14^. Impaired mitochondrial biogenesis and functions in adipose tissue are also observed in animal models of type 2 diabetes^15^. Furthermore, a decrease in mitochondrial mass and function has been found in adipose tissue of obese ob/ob mice^16^. However, the impact of excessive ADM found in GDM patients on adipocyte mitochondrial function remains unclear. In the present study, we hypothesized that excessive ADM may induce adipocyte mitochondrial respiratory dysfunction and contributes to adipocyte-related metabolic complications. To address this hypothesis, we studied the impact of ADM on mRNA expression of mitochondrial DNA (mtDNA)-encoded subunits of electron transport chain, mitochondrial content, reactive oxygen species (ROS) generation, and mitochondrial respiratory capacity in human adipocytes.

## Results

### Glucose suppresses mRNA expression for mtDNA-encoded subunits ND1 and ND2 in electron transport chains.

**Figure 1:**
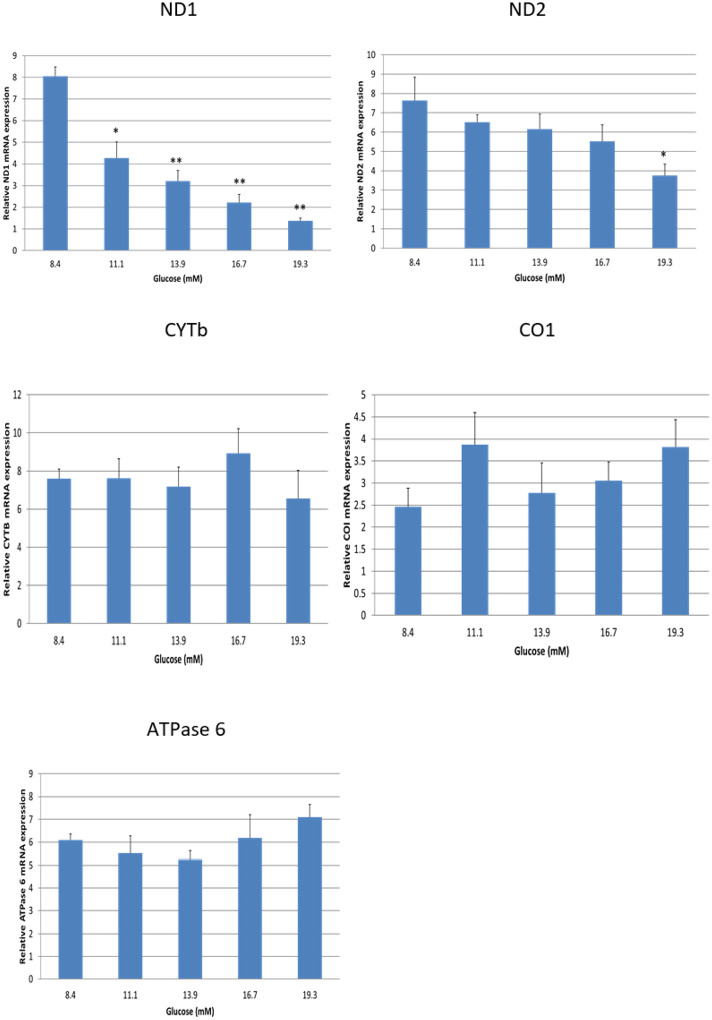
Glucose dose-dependently inhibits mRNA expressions of mtDNA-encoded subunits of ND1 in electron transport chain in human adipocytes. Data are presented as the mean ± SEM from three repeated experiments. *p < 0.05, **p < 0.01 compared with the untreated controls.

To investigate the effect of increasing doses of glucose on mtDNA-encoded subunits in the electron transport chain in human adipocytes, we measured the gene expression for mtDNA-encoded subunits of the electron transport chain using q-PCR. As shown in Fig. 1, the expressions of ND1 were significantly inhibited in the adipocytes by glucose in a dose dependent manner (P < 0.01), and ND2 was down regulated at higher dose of glucose (P < 0.05). However, the alterations in mRNA expression for CYTb, CO1, and ATPase 6 were not significant compared with controls (P > 0.05). These results indicate that increased glucose concentration, mimicking the hyperglycemia environment in GDM patients, is associated with reduced mtDNA-encoded subunits of the electron transport chain in human adipocytes.

### ADM inhibits mRNA expression for mtDNA-encoded subunits ND1, ND2, CYTb, and ATPase in the adipocytes.

**Figure 2:**
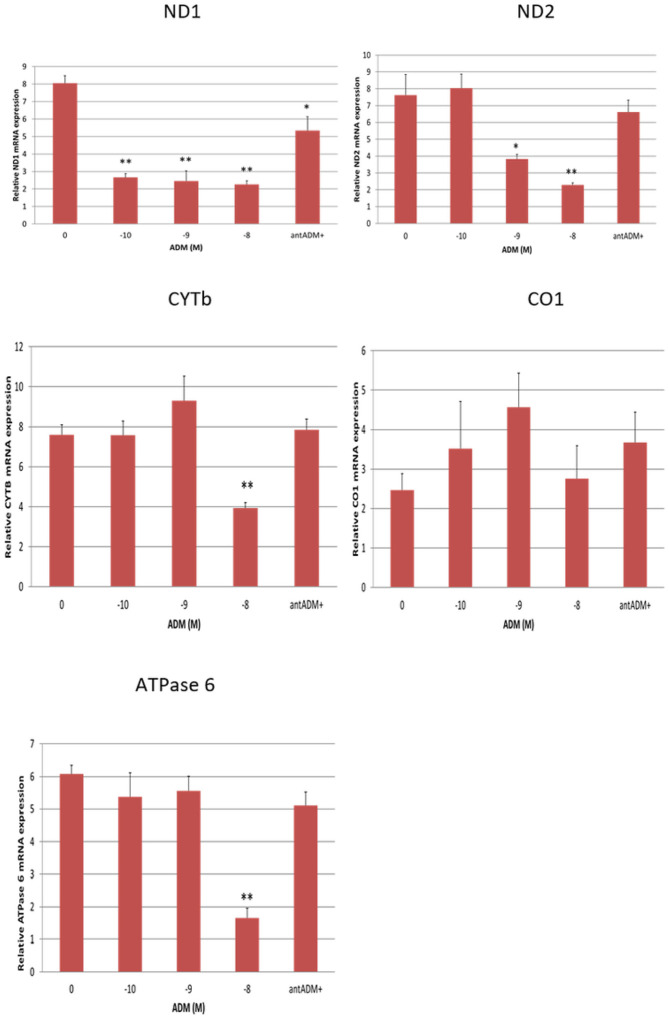
ADM inhibits mRNA expressions of mtDNA-encoded subunits of electron transport chain, ND1, ND 2, CYTb, and ATPase 6 in human adipocytes. Data are presented as the mean ± SEM from three repeated experiments. *p < 0.05, **p < 0.01 compared with the untreated controls.

To investigate the impact of ADM on mtDNA-encoded subunits in human adipocytes, we treated the cells with increasing dose of ADM for 24 hours. As shown in Fig. 2, mRNA levels for ND1, ND2, CYTb, and ATPase (P < 0.05 or P < 0.01), but not CO1 (P > 0.05), were inhibited by ADM in the adipocytes. Moreover, coincubation with ADM antagonist, ADM22–52, blocked the effects of ADM indicating the specificity of ADM effects. This finding suggests that excessive ADM expression seen in adipose tissue from GDM patients may induce a decrease in mRNA levels for mtDNA-encoded subunits of the electron transport chain.

### ADM does not significantly affect adipocyte mitochondrial content.

**Figure 3:**
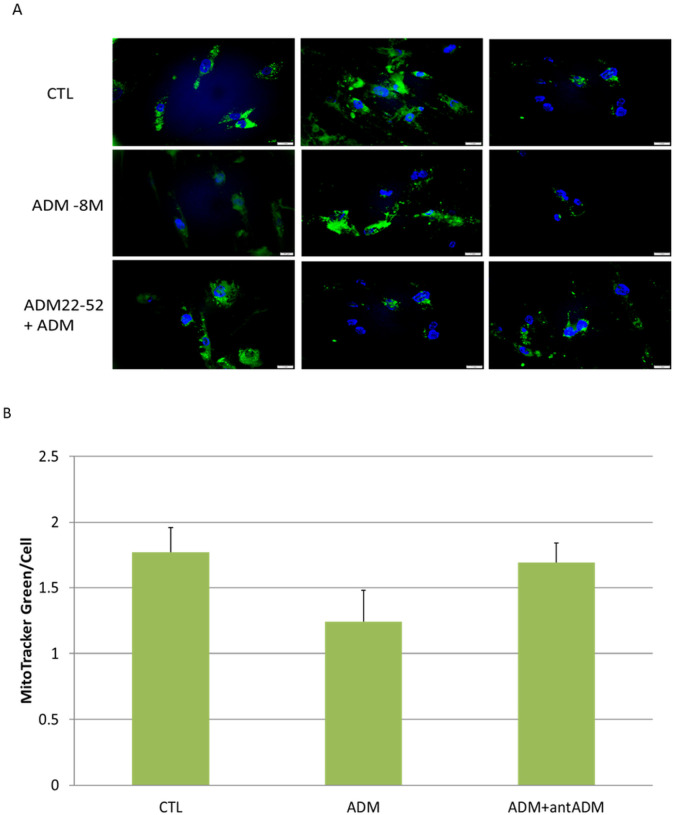
Representative images showing the mitochondrial contents by using mitochondrial-specific fluorescence MitoTracker green (A), and the analysis of intensity per cell (B). No significant difference was noted between the groups. Data are presented as the mean ± SEM from three repeated experiments.

The number of copies of mtDNA per cell is a general marker of mitochondrial fitness. To provide further evidence of the mitochondria regulation by ADM in adipocytes, we determined the mitochondrial content by staining the cells with Mitochondrial-specific fluorescence dye MitoTracker green. As shown in Fig. 3, adipocytes treated with ADM were weakly stained with MitoTracker compared with controls, and ADM antagonist ADM22–52 partially reverse the reduced staining, but no significant differences were detected between group, implying that ADM does not significantly affect mitochondrial content in human adipocytes.

### ADM induces ROS generation in adipocytes.

**Figure 4:**
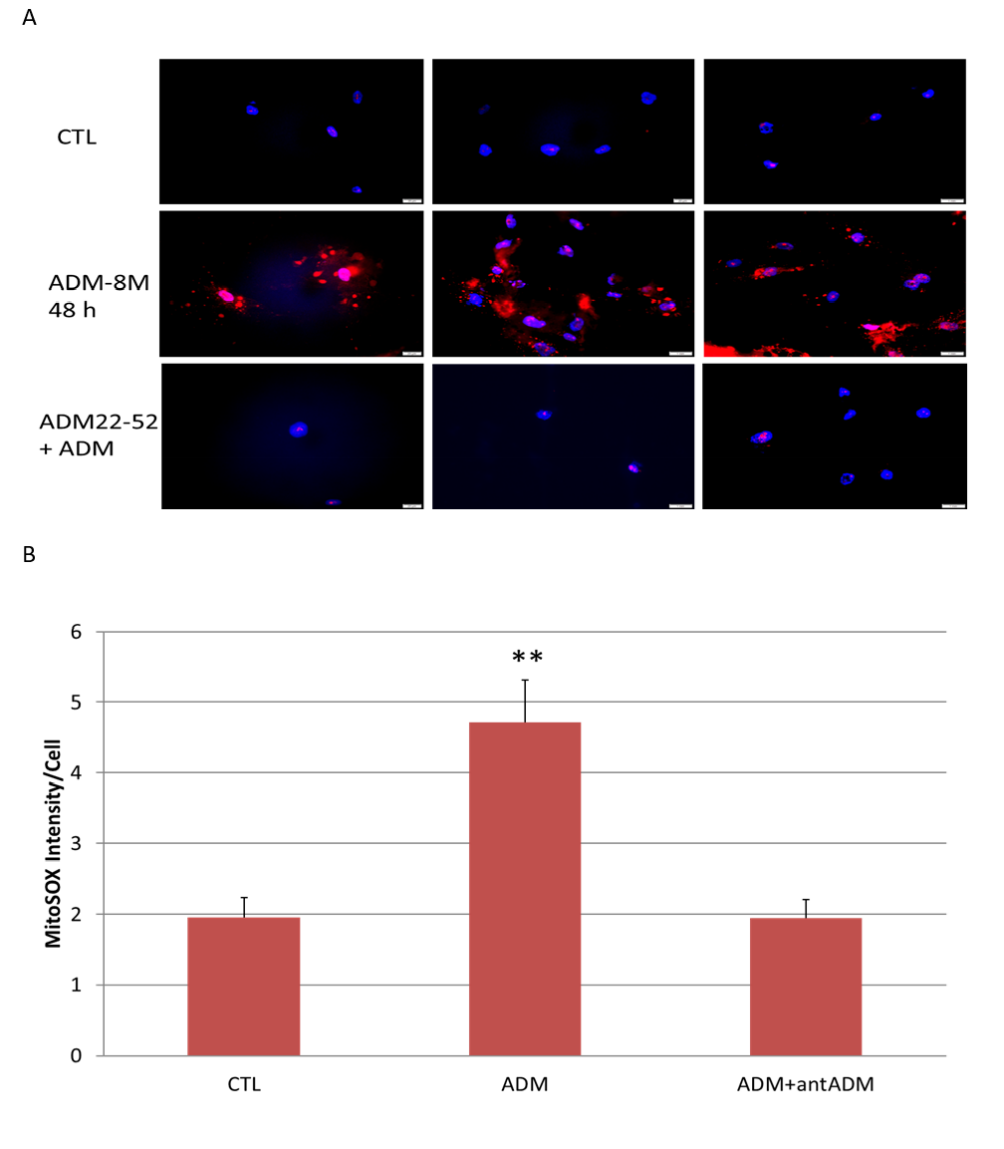
Representative images showing the reactive oxygen species (ROS) by using MitoSOX red probe (A) and the analysis of intensity per cell (B). ADM (−8M) stimulates ROS expression in human adipocytes, and this increase was blocked by ADM antagonist, ADM22–52 (−7M). Data are presented as the mean ± SEM from three repeated experiments. **p < 0.01 compared with the untreated control.

ROS, the by-products of mitochondrial respiration, are produced normally by the adipocytes, and overproduction of the ROS may damage various components in the cells. To evaluate the effect of ADM on ROS generation, we measured ROS levels in adipocytes by MitoTracker Red, a mitochondrion-specific dye. As shown in Fig. 4, ADM stimulates ROS production in adipocytes as compared to controls, and this increase was reversed by ADM antagonist, ADM22–52. These results suggest that ADM increases adipocyte ROS generation, and this increase is specific to ADM, which may contribute to adipocyte systemic mitochondrial dysfunction.

### ADM disrupts mitochondrial respiratory capacity.

**Figure 5:**
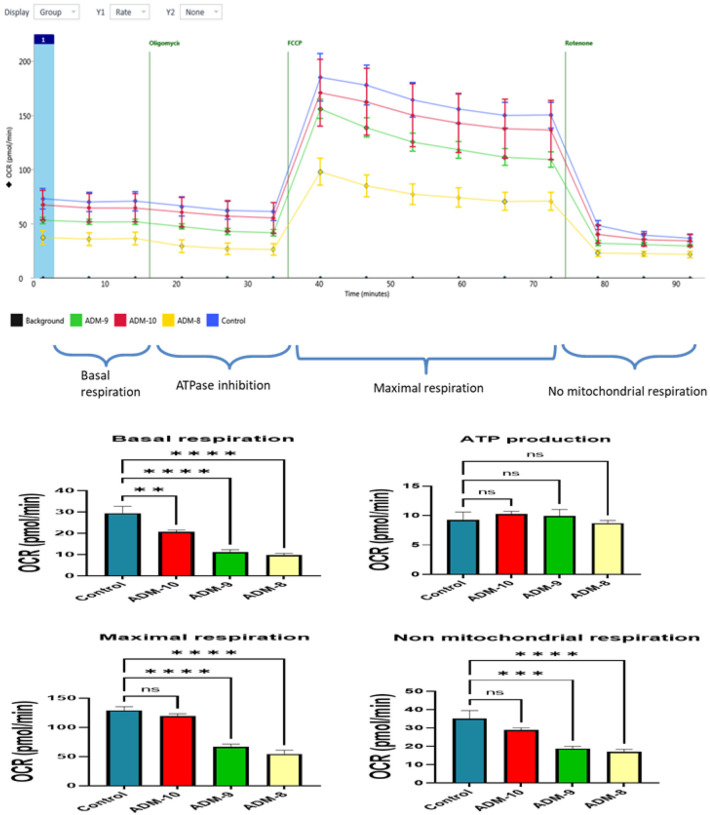
ADM suppresses adipocyte basal, maximal and non-mitochondrial oxygen consumption rate in a dose-dependent manner. (A) A typical bioenergetics profile involving a four-step analysis. (B) Basal oxygen consumption rate (OCR); (C) ATP production; (D) Maximum respiration, and (E) Non mitochondrial respiration. Data are presented as the mean ± SEM from 6 repeated samples. ** *p* < 0.01, ****p < 0.001 compared with the untreated controls.

To further test the effects of ADM on mitochondrial function, we assessed mitochondrial respiratory capacity using the Seahorse Biosciences XF-96 Analyzer. We used a typical bioenergetic profile, involved in a four-step analysis: (1) basal OCR, adipocytes were incubated in normal medium; (2) ATP synthesis turnover, oligomycin (2.0 mM) was supplemented to the medium to inhibit ATP synthase; (3) maximal mitochondrial respiratory capacity, cells were motivated with FCCP (1.0 mM); and (4) non-mitochondrial respiration, rotenone (1.0 mM) was introduced to inhibit complex I. As shown in Fig. 5, increasing doses of ADM (−10 to −8M) exerted negative effects on the mitochondrial respiratory function of human adipocytes. Specifically, the basal mitochondrial respiration was inhibited by ADM starting from concentration of −10 M, and further reduced by ADM at a concentration of −9M and − 8M (P < 0.01). Moreover, ADM also inhibited the maximal mitochondrial and non-mitochondrial OCR of adipocytes in a dose-dependent manner (P < 0.01). However, there was no significant changes in the ATP linked OCR of the cells treated with ADM (P > 0.05). These results indicate that ADM significantly suppresses mitochondrial respiratory function, denoting reduced ability of mitochondria to respond to increased energy requirements, but ATP linked OCR was not significantly affected.

## Discussion

Mitochondria are important organelles participating in the regulation of numerous cellular activities, including thermogenesis, ROS generation, redox and Ca^2+^ homeostasis, and cell apoptosis. Mitochondrial dysfunction in adipocytes can affect whole-body energy homeostasis as well as insulin resistance^17^. In the present study, we performed a comprehensive set of experiments to test a hypothesis that ADM impaired mitochondrial function in human adipocytes. Our data revealed that both glucose and ADM inhibit human adipocytes mRNA expressions of mtDNA-encoded subunits of electron transport chain, including ND 1 and 2, CYTb, and ATPase 6. Furthermore, ADM stimulates mitochondrial ROS generation, but does not affect the mitochondrial contents in the adipocytes. In addition. ADM suppresses adipocyte basal and maximal oxygen consumption rate in a dose-dependent manner, leading to compromised mitochondrial respiratory capacity. Therefore, excessive ADM seen in GDM patients may contribute to lipid metabolic dysregulation through disrupting adipocyte mitochondrial function. Thus, these data bring new insights into GDM-related adipose tissue dysfunction.

Mammalian mitochondria possess their own genome, which consists of a single, circular double-stranded mtDNA molecule^18^. mtDNA encodes essential components of complexes of the electron transport chain, including (1) Complex I: seven nicotinamide adenine dinucleotide dehydrogenase subunits involved (ND1, ND2, ND3, ND4L, ND5 and ND6) of NADH dehydrogenase; (2) Complex III: the cytochrome b (CYTb) subunit of the ubiquinol-cytochrome c oxidoreductase involved; (3) Complex IV: three subunits (COI, COII and COIII) of cytochrome c oxidase involved, and (4) Complex V: the ATPase 6 and 8 subunits, which are necessary for protein production within the mitochondria^19^. It has been reported that decreased expression of the genes in complexes I and IV leads to adipocyte dysfunction^20^, and reduced mRNA for complex I, III and V can induce triglyceride (TG) accumulation in 3T3-L1 cells^21^. Present study demonstrated that ADM dose-dependently inhibited human adipocyte mRNA expressions of mtDNA-encoded subunits of electron transport chain, including ND1 and 2, CYTb, and ATPase 6, suggesting the negative impact of ADM on adipocyte mitochondrial function. Considering mitochondrial mtDNA impairment is associated with reduced fatty acid-oxidation and increased cytosolic free fatty acid accumulation in adipocytes that alters glucose uptake^22^, our results may reveal a novel molecular mechanism linking adipocyte-ADM and mitochondrial dysfunction in the pathogenesis of diabetic pregnancy.

The new mitochondria generation involves complete replication of mitochondrial DNA. Mitochondrial biogenesis is driven by the transcriptional activator of NRF-1, NRF-2, PGC-1α, which is activated by various pathways such as receptor tyrosine kinases, natriuretic peptide receptors and nitric oxide through the generation of cGMP^23^. It has been reported that both mitochondrial mass and respiratory chain activity are decreased in adipocytes in diabetic mice^15^, implying impaired mitochondrial biogenesis by glucose dysregulation. Our data showed that ADM does not significantly alter the content of mitochondria in human adipocytes, thus the impaired mitochondrial function in the adipocyte is unlikely resulted from a lower number of mitochondria mass, at least in our present study.

Mitochondrial ROS are generated by the respiratory chain, and thus indirectly associated with the status of mitochondrial activity. Evidence have proven that low concentrations of ROS functions as secondary messengers, playing a role in cell signaling inside and outside mitochondria^16^. However, excessive mitochondrial ROS generation in adipocytes by chronic oxidative stress may contribute to the development of insulin resistance and the progression of various metabolic diseases, including GDM. Particularly, increased ROS production in 3T3-L1 preadipocytes has been demonstrated to be associated with inhibited cell proliferation^24^, and elevated intracellular ROS levels impair adipocyte function, which is accompanied by glucose intolerance and insulin resistance^25^. GDM is associated with higher ROS generation compared with normal pregnancies^26^. In the present study, we used cultured adipocytes to assess the effects of ADM on ROS generation. The MitoSOX^™^ Red staining, the superoxide indicator of mitochondria, were significantly enhanced in ADM treated adipocytes compared with controls, indicating that oxidative stress was induced by ADM in adipocytes, thus, increased circulating ADM in GDM patients may contribute to the metabolic complications, including glucose intolerance and insulin resistance.

Mitochondrial respiratory capacity is vital to the functionality and viability of the adipocytes, and cellular oxygen consumption is a fundamental indicator of mitochondrial function. Specifically, mitochondrial basic respiration includes coupled as well as uncoupled mitochondrial oxygen consumption^27^. The coupled oxygen consumption produces ATP, and the uncoupled oxygen consumption forms ROS, which is involved in multiple physiological and pathological activities. In addition, the maximal OCR is an indicator which represents the ability of mitochondria to reserve energy^28^, and mitochondrial stress often leads to excessive ROS generation and mitochondrial dysfunction. The present study revealed that ADM induces mitochondrial stress by inhibiting basal and maximal mitochondrial OCR in a dose-dependent manner. Accordingly, non-mitochondrial respiratory capacity, roughly displaying adaptation to metabolic changes, was also reduced by ADM in adipocytes. On the contrary, no significant differences in ATP linked OCR were detected between groups, indicating that oligomycin addition had no significant impact on ADM treated adipocytes. It has been reported that mitochondrial ATP is generated from reduced equivalent electron carrier nicotinamide adenine dinucleotide (NADH or NAD + H+) (complex I, NADH dehydrogenase) and reduced flavin adenine dinucleotide (FADH2) (complex II, succinate dehydrogenase), and finally through oxidative phosphorylation at the F0F1-ATP synthase (complex V)^27^. In the present study, although we found the expression of ND1, ND2, CYTb, and ATPase 6 were inhibited by ADM in adipocytes, but the role of other parts of Complex I and V in the balance of the ATP production and consumption remains unclear. Thus, further study focusing on the mRNA, proteins, and activity for mtDNA-encoded subunits of electron transport chain, including but not limited to ND3, ND4, ND4L, ND5 and ND6 of NADH dehydrogenase and ATPase 6 and 8, are apparently warranted.

In conclusion, our findings provide evidence of ADM treatment resulted in mitochondrial dysfunction in human adipocytes, and excessive ADM found in GDM patients may act as a circulating factor linking energy generation and consumption and contribute to impaired adipocyte mitochondrial metabolism in diabetic pregnancy. Therefore, the new concept that ADM regulates mitochondrial functions may have therapeutic potential for the treatment of important pathophysiological conditions related to glucose/lipid metabolism.

### Limitation:

The influence of ADM on the activity of complex I, III, IV, and V in the mtDNA needs to be clarified. In addition, the specific downstream signaling underlying ADM effects on mitochondrial biogenesis and the ex vivo effects of ADM and its antagonist on the mitochondrial biogenesis and function in adipose tissue from GDM patients remain to be explored.

## Materials And Methods

### Human pre-adipocyte culture

Primary normal human pre-adipocytes (ATCC PCS-210–010, American Type Culture Collection, Manassas, VA, USA) were differentiated into mature adipocytes in wells of 24-well-plates containing adipocyte differentiation medium (Cell Applications, Inc. San Diego, CA) in a 5% CO2 atmosphere at 37^0^ C^29^. These cells can be expanded in an undifferentiated state for future differentiation to mature adipocytes and show higher efficiency of adipogenesis compared to mesenchymal stem cells. In this study, the cells were cultured in adipocyte differentiation medium with increasing doses of glucose (8.4mM to 19.3mM, Sigma-Aldrich, St. Louis, MO), or ADM (1×10^− 10^M to 1×10^− 8^M, Sigma-Aldrich) for 24 hours. Total RNA was isolated from the cells using TRIzol (Life Technologies, Grand Island, NY) and RT was performed for further Quantitative Real-time-PCR analysis.

### The mRNA expression for mitochondrial DNA (mtDNA)-encoded subunits of the electron transport chain

Quantitative Real-time-PCR was performed using Taq universal SYBR Green Supermix (Bio-Rad). PCR primers used for amplification for mitochondrial DNA (mtDNA)-encoded subunits of the electron transport chain were purchased from Integrated DNA Technologies (IDT) and the primer sequences were listed in Table 1. Amplification of 18S and GAPDH served as endogenous controls. PCR conditions for SYBR Green gene expression were 10 min at 95°C for 1 cycle, then 15 sec at 94°C, 30 sec at 60°C and 15 sec at 72°C for 39 cycles. All experiments were performed in triplicate. The average CT value was used to calculate the results using the 2–ΔΔCT method and expressed in fold increase/decrease of the gene of interest.

### Measurement of mitochondrial contents

Human pre-adipocytes were seeded onto 8 chamber glass slides containing adipocyte differentiation medium, differentiated adipocytes were treated with ADM (1×10^− 8^M) with or without ADM22–52 (1×10^−7^M) for 48 h. The cells were then loaded with Mitochondrial-specific fluorescence dye MitoTracker green (100 nM, Invitrogen) for 45 min at 37°C. The slides were then mounted with mounting-medium containing 4′, 6-diamidino-2-phenylindole (DAPI; Vector Laboratories Inc., Burlingame, CA) and viewed under an Olympus BX51 microscope. The intensity of the immunofluorescence was measured by using CellSence software (Olympus Scientific, Walthan MA, USA), and the relative densities of the immunofluorescence to the number of nuclei were calculated and compared between groups.

### Assessment of mitochondrial reactive oxygen species (ROS)

Human pre-adipocytes were seeded onto 8 chamber glass slide containing adipocyte differentiation medium. Differentiated adipocytes were treated with ADM (1×10^− 8^M) with or without ADM22–52 (1×10^− 7^M) for 48 h. The cells were then loaded with MitoSOX red probe (5 μM, Invitrogen) for 10 min at 37°C. The slides were then mounted with mounting-medium containing 4′, 6-diamidino-2-phenylindole (DAPI; Vector Laboratories Inc., Burlingame, CA) and viewed under an Olympus BX51 microscope. The intensity of the immunofluorescence was measured by using CellSence software (Olympus Scientific, Walthan MA, USA), and the relative densities of the immunofluorescence to the number of nuclei were calculated and compared between groups.

### Determination of the mitochondrial oxygen consumption rate (OCR)

Ten thousand preadipocytes per well were seeded in 96-well XF assay plates containing adipocyte growth medium and differentiated into mature adipocytes in the presence or absence of ADM (1×10^− 10^M to 1×10^− 8^M). The cells were then subjected to real-time measurements of oxygen consumption rate (OCR) using Seahorse Biosciences XF-96 Analyzer (Agilent, CA). For mitochondrial stress tests, mitochondrial complex inhibitors were injected to all the following treatments sequentially in the following order: oligomycine (1.5 μM), carbonyl cyanide-ptrifluoromethoxyphenylhydrazone (FCCP; 0.5 μM), antimycine A/rotenone (0.5 μM each), and 3 readings were taken after each injection. OCR was automatically recorded by XF-96 software provided by the manufacturer. Calculations of proton leak, coupling efficiency, and maximal respiration were performed according to the manufacturer’s instructions.

### Statistics

All data were presented as mean ± SEM. Data were calculated and analyzed by GraphPad Prism (La Jolla, CA). Repeated measures ANOVA (treatment and time as factors) with a Bonferroni post hoc test were used for comparisons between groups. mRNA and protein expression were compared between control and treatment groups using unpaired Student t test. Statistical significance was defined as p < 0.05.

## Figures and Tables

**Table 1.** Primer sequence for RT-PCR

	Forward	Reverse
ND1	5′-GGGCTACTACAACCCTTCGCT-3′	5′-GAGGCCTAGGTTGAGGTTGAC-3′
ND2	5′-CACAGAAGCTGCCATCAAGTA-3′	5′-CCGGAGAGTATATTGTTGAAGAG-3′
CYTb	5′-TCATCGACCTCCCCACCCCATC-3′	5′-CGTCTCGAGTGATGTGGGCGATT-3′
CO1	5′-TCATGATCACGCCCTCATA-3′	5′-CATCGGGGTAGTCCGAGTAA-3′
ATPase 6	5′-GCCCTAGCCCACTTCTTACC-3′	5′-TTAAGGCGACAGCGATTTCT-3′
18S	5′-TTCGAACGTCTGCCCTATCAA-3′	5′-ATGGTAGGCACGGCGACTA-3′
GAPDH	5′-GGTCTCCTCTGACTTCAACA-3′	5′-AGCCAAATTCGTTGTCATAC-3′

Complex I: nicotinamide adenine dinucleotide dehydrogenase (ND1 and ND2);

Complex III: cytochrome b (CYTb);

Complex IV: cytochrome c oxidase (CO1);

Complex V: ATPase 6 and 8 subunits (ATPase 6).

## Data Availability

The datasets used and/or analyzed during the current study available from the corresponding author on reasonable request.
